# Analysis of Transcriptome and Differentially Expressed Genes in Chicken Primordial Germ Cells

**DOI:** 10.3390/ani16030522

**Published:** 2026-02-06

**Authors:** Anastasiia I. Azovtseva, Anna E. Ryabova, Artem P. Dysin, Grigoriy K. Peglivanyan, Natalia R. Reinbach, Alina V. Gabova, Olga Y. Barkova, Ekaterina A. Polteva, Tatiana A. Larkina

**Affiliations:** Russian Research Institute of Farm Animal Genetics and Breeding (RRIFAGB)—Branch of the L.K. Ernst Federal Science Centre for Animal Husbandry, Pushkin, 196601 St. Petersburg, Russia; aniuta.riabova2016@yandex.ru (A.E.R.); artemdysin@mail.ru (A.P.D.); peglivanian_grig@mail.ru (G.K.P.); miss.reynbax@yandex.ru (N.R.R.); alina.gabova7@yandex.ru (A.V.G.); barkoffws@list.ru (O.Y.B.); ketlin.liselse@yandex.ru (E.A.P.)

**Keywords:** transcriptome, DEGs, chicken, *Gallus gallus domesticus*, primordial germ cells, genome editing, CRISPR/Cas9, gene expression, differential expression analysis

## Abstract

Achieving successful primordial germ cell (PGC)-based genome editing requires a deep understanding of their molecular identity. For the first time, a comparative transcriptomic analysis of chicken PGCs and adult liver cells to define their specific gene expression signature was performed. PGCs were isolated from Rhode Island Red chicken embryos, cultured, and subjected to RNA sequencing alongside liver tissue. Differential expression analysis with Benjamini–Hochberg correction identified 1909 differentially expressed genes (DEGs). Functional annotation revealed that PGCs possess a unique transcriptional landscape, characterized not only by enhanced proliferation and metabolic activity but also by a profound molecular convergence with neural crest cells. This is evidenced by the upregulation of gene modules governing long-range migration, neuronal signaling, and specialized “neuro-lipid” metabolism (e.g., sphingolipid and plasmalogen pathways). Additionally, we identified unannotated transcripts linked to immune pathways and ciliary signaling. Our study expands the functional annotation of avian PGCs and reveals an unexpected evolutionary recruitment of conserved morphogenetic programs, providing a refined molecular foundation for advanced germline editing technologies.

## Simple Summary

Successful genome editing using primordial germ cells requires a detailed understanding of their functioning during embryonic development. In this study, we performed, for the first time, a comparative transcriptome analysis between chicken primordial germ cells and adult liver cells. We identified a total of 1909 differentially expressed genes involved in intracellular metabolism and protein biosynthesis, including transcription, translation, and post-translational protein modifications. This expression signature is consistent with the highly active and undifferentiated state of primordial germ cells during early embryogenesis. The findings provide a valuable resource for understanding gene activity within avian germ cells and establish a foundational transcriptomic signature for primordial germ cells. This knowledge could potentially serve as an important tool for maintaining the genetic biodiversity of unique, small, and endangered chicken populations and breeds.

## 1. Introduction

Qualitative breakthroughs in the development of genome editing (GE) tools and technologies allow for precise genomic modifications [1], demonstrating high potential for the development of biomedical, biotechnological and agricultural industries [2,3]. GE-technologies enable targeted GE for both the treatment of human diseases [4] and the improvement in productive traits and the achievement of desired phenotypic functions in livestock, including disease-specific resistance [2,5]. Current mammalian GE-protocols mainly involve embryo editing with its subsequent transplantation to an animal recipient. However, this approach is not feasible in avian species due to their embryogenesis peculiarities [1]. Despite the lack of successful transfer of genetic modifications when editing chicken embryos in ovo [3], studies exploring GE in chickens persisted. A pivotal solution came with the development of the in vitro cultivation protocol for primordial germ cells (PGCs), facilitated by the identification of PGC self-renewal mechanisms [6,7]. PGCs are gamete precursors, the only cell type capable of transmitting genetic and epigenetic information across generations [8,9]. Chicken PGCs originate from the epiblast during early embryogenesis [10], when the germ cell line separates from the somatic cell line [11]. They are first identified in the center of the area pellucida (stage X blastoderm) [12], from where they migrate to the germinal crescent [13]. Subsequently, PGCs enter the vascular system [14] and use it to reach the germinal ridges, where they accumulate as gonadal PGCs or gonocytes [15]. Later, they differentiate into spermatogonia and oogonia [9].

PGCs have great potential for genome editing. Their capacity for in vitro cultivation allows for genetic modification and reproduction in clonal populations, which can be used for creating chimeras capable of producing offspring with a modified germ cell line [1,3]. The advantage of PGCs is their availability at many embryonic stages; they can be isolated by both embryo dissection and blood aspiration [3]. The latter method is feasible because, unlike most mammals, chicken PGCs migrate to the gonadal buds via the vascular system instead of the intestinal epithelium [14,16]. Ultimately, PGCs can significantly improve the quality of agriculture and livestock products by the rapid introduction of desired phenotypic traits, improving disease management, enhancing animal welfare, and ensuring the safety of chicken egg-grown vaccines [1]. A significant contribution can be made to the conservation of global biodiversity. Intensive selection has reduced the genetic diversity of bird species almost by half [6,17], creating an urgent need for effective ex situ conservation strategies [18]. Cryopreservation of germplasm faces a fairly low efficiency in birds due to the polylecithal egg structure and the reduced fertility of cryopreserved semen [19]. In this context, PGCs biobanking is a promising alternative for preserving biomaterial from endangered species for future population recovery [20,21].

However, the successful application of PGC-based genome editing requires substantial information on the embryonic development governing germ cell production from PGCs, alongside knowledge on the peculiarities of PGC functioning. The latter include migration, survival, sexual differentiation, and epigenetic reprogramming [11].

The aim of the present study was to perform a comparative transcriptome analysis of chicken PGCs and adult liver cells. Through differential gene expression analysis, we aimed to identify key genes active in PGCs and to expand the existing knowledge of their functional genomics.

## 2. Materials and Methods

### 2.1. Animal Sampling

Biological material. PGCs (*n* = 2) were obtained from embryos of the Rhode Island Red chicken breed, kept in the Center of Collective Use (CCU) "Genetic Collection of Rare and Endangered Chicken Breeds" (Pushkin, Saint-Petersburg, Russia). As a control, liver cells (*n* = 2) were obtained from adult roosters of the same breed via post-mortem cell sampling at the age of 52 weeks.

Rationale for control tissue selection. Adult liver was selected as a negative control tissue for transcriptomic comparison. Liver cells represent a terminally differentiated somatic state with a stable metabolic profile. This choice provides a maximal transcriptional contrast to the pluripotent, migratory PGCs, thereby allowing the filtering out of ubiquitous housekeeping gene expression and facilitating the robust identification of genes that are specifically and highly upregulated in the germ cell lineage.

### 2.2. PGC Culture Medium

Culture medium for PGC cultivation consisted of base medium Opti-MEM (Reduced Serum Medium, GlutaMAX^TM^ Supplement) (Gibco^TM^, Thermo Fisher Scientific Inc., Waltham, MA, USA) supplemented with sodium pyruvate (Applichem GmbH., Darmstadt, Germany)—1 M; EmbryoMax nucleosides (100×) (MilliporeSigma, Burlington, MA, USA)—up to 1×; chicken serum (Gibco^TM^, Thermo Fisher)—2%; 2-mercaptoethanol (NF grade, VWR International, Radnor, PA, USA)—0.01%; antibiotic-antimycotic (100×) (Gibco^TM^, Thermo Fisher)—up to 1×; human Activin A Recombinant Protein (Gibco^TM^, Thermo Fisher)—25 ng/µL; human FGF-basic (FGF-2/bFGF) Recombinant Protein (Gibco^TM^, Thermo Fisher)—10 ng/µL.

### 2.3. Derivation and Culturing of Chicken PGCs

**Figure 1:**
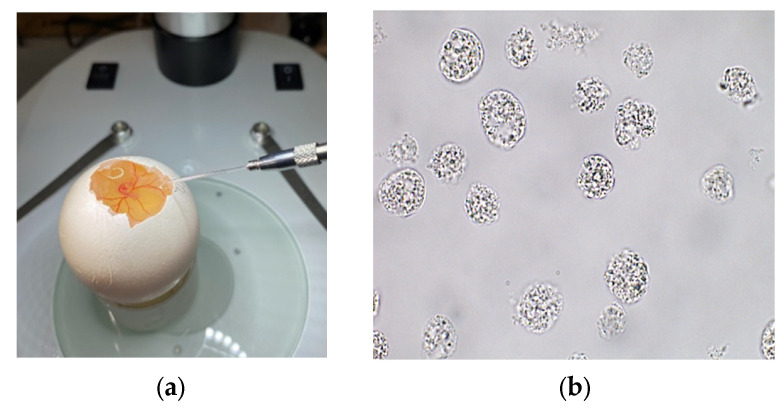
Blood sample collection for PGC extraction via microinjector (**a**) and PGC culture at 21st day of cultivation (**b**).

Fertilized eggs from the Rhode Island Red chicken breed were screened and subsequently disinfected. Egg incubation was carried out up to stage 13–14 by Hamburger-Hamilton (approximately 50–52 h post-laying) [22]. At this stage, PGCs are bipotent and have not initiated sex-specific differentiation programs. PGC extraction was performed using embryo blood. Blood samples were collected by egg dissection using a graver (Dremel Inc., Mount Prospect, IL, USA), namely by opening a small window (approximately 10–15 mm in diameter), without damaging the underlying shell membrane. Blood was aspirated from the dorsal aorta of the embryos using a microinjector (model IM-11-2, Narishige Co., Ltd., Tokyo, Japan) (Figure 1a). The collected blood samples were immediately transferred to prepared vials with a culture medium and maintained at +37 °C until plating. PGC cultivation was performed in multiwell plates with supplemented culture medium and growth factors at +37 °C for 21 days (Figure 1b). Quantification of PGC cultures started within 10–12 days of cultivation.

We did not perform genetic sex determination of the pooled PGC population prior to RNA-seq analysis. Therefore, the PGC samples represent a mixed population potentially containing cells from both male and female embryos.

### 2.4. RNA Extraction and Library Preparation

RNA isolation was performed using the MagMax^TM^ Total RNA Isolation Kit (Thermo Fisher Scientific Inc., Waltham, MA, USA). RNA quality assessment was performed by quantitative fluorescence and fragment length analysis, yielding the RNA integrity number (RIN) and DV200 index to evaluate the degree of degradation and fragmentation. Sequencing libraries were performed using the TruSeq Stranded mRNA Library Prep Kit (Illumina Inc., San Diego, CA, USA). To minimize batch effects and ensure comparability, all the samples (both PGC and liver) underwent RNA extraction, library preparation, and sequencing in the same batch, under identical protocols and on the same sequencing lane of the NovaSeq 6000 platform.

### 2.5. Generation of RNA-Seq Data and Bioinformatics Analysis

Sequencing was performed using the NovaSeq 6000 platform (Illumina Inc., San Diego, CA, USA), with read length—2 × 101 b.p. Quality control revealed that 92.06% of the reads had a Phred quality score of Q30 (probability 1 wrong base per 1000 readings). Demultiplexing of the raw data was performed using bcl2fastq2 (v2.20) (Illumina Inc., San Diego, CA, USA), and removal of adapter sequences with Skewer (v0.2.2b) [23]. The trimmed reads were aligned to the reference genome of Red Jungle Fowl (GRCg6a assembly) using the STAR aligner (v2.7.3) [24].

### 2.6. Transcriptome Comparison and Differential Gene Expression Analysis

Differential expression analysis between groups was performed using the DESeq2 package (v1.24.0) in the R (v4.0.4) [25] programming environment. DESeq2 uses a negative binomial generalized linear model to test for differential expression based on raw gene counts.

According to DESeq2 recommendations, the genes with fewer than two reads were removed from all the samples, as their variance is the highest and they often give false positives. The package internally performs size factor normalization to account for differences in library depth. Using the normalized counts, DESeq2 calculates the log_2_ fold changes (log_2_FC) and tests their statistical significance using the Wald test. The resulting *p*-values were adjusted for multiple testing using the Benjamini–Hochberg correction to control the false discovery rate (FDR) [26]. The genes with log_2_ FC > 1.5 and an adjusted *p*-value (padj) < 0.05 were marked as significantly differentially expressed.

To assess the overall sample relationship, we performed hierarchical clustering and principal component analysis (PCA). These visualizations required the application of the variance-stabilizing transformation (VST) to the count data to ensure equal gene contribution in the distance between the samples.

The Gene Ontology (GO) enrichment analysis for biological processes was conducted on the identified DEGs using the GO terms from the GO database (URL: https://geneontology.org/ accessed on 23 September 2025) using the clusterProfiler package (v4.3.1.900) in R (v4.0.4). Enrichment *p*-values were also corrected for multiple testing using the Benjamini–Hochberg correction. The results were visualized using ggplot2 (v3.4.4) and ggdendro packages (v0.1.22) in R (v4.0.4). Functional annotation of individual genes was performed via the NCBI genome browser.

## 3. Results

RNA sequencing was conducted on a total of four samples for differential expression analysis. Group 1 contained two liver cell samples, and Group 2—two PGC samples. The quality metrics and output statistics of the obtained raw data are presented in Table 1.

**Figure 2:**
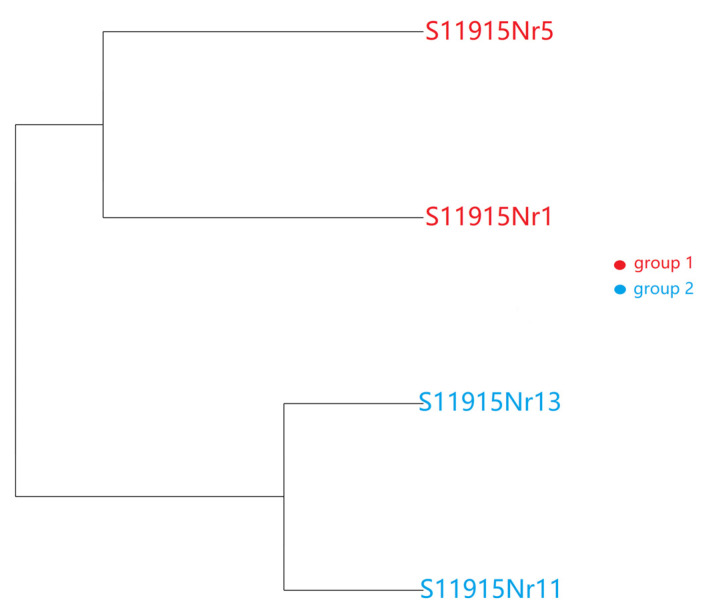
The dendrogram from hierarchical cluster analysis of gene expression data. Samples from adult liver (Group 1) and PGCs (Group 2) are indicated.

The dendrogram revealed clear separation between the two cell types, with sample clustering exclusively by their origin (Figure 2). This underscores their transcriptomic differences.

**Figure 3:**
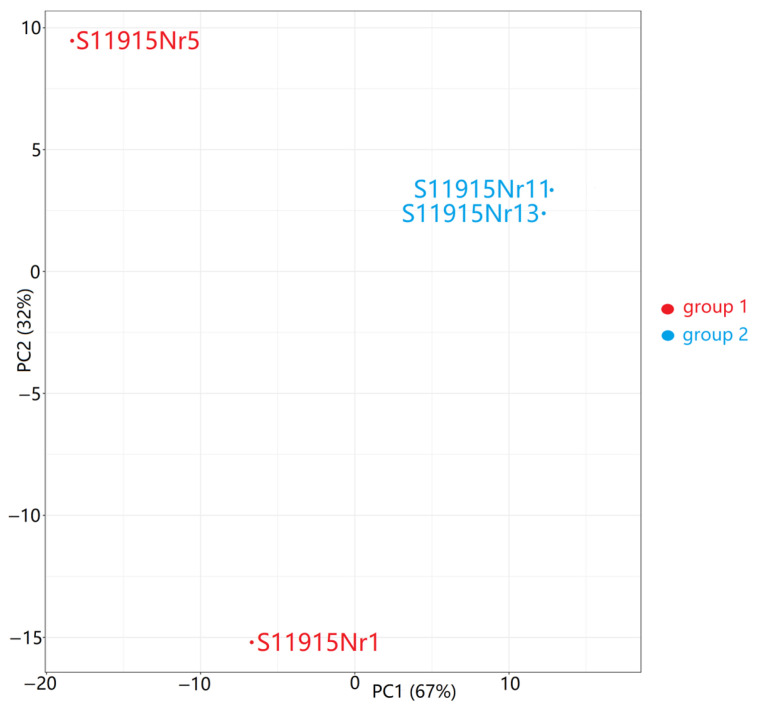
Principal component analysis (PCA) of transcriptome data. The plot shows the separation between the samples of adult liver (Group 1) and PGCs (Group 2) based on variance-stabilized gene expression values.

Differential expression analysis between Group 2 (PGCs) and Group 1 (liver cells) was performed on a total of 16,859 genes (Table S1). Principal component analysis (PCA) was applied to visualize the transcriptomic relationships (Figure 3). The first (PC1) and second (PC2) components explained 67% and 32% of the variance between the samples, respectively. The observed PGC sample clustering indicates a high similarity in their sets of differentially expressed genes. In contrast, the liver cell samples did not show any clustering; sample S11915Nr1 had moderate negative loading on PC1 and high negative loading on PC2, whereas sample S11915Nr5 showed high negative loading on PC1 and high positive loading on PC2. The notable heterogeneity among the liver samples most likely reflects the natural physiological variation and complex functional state of the adult liver.

**Figure 4:**
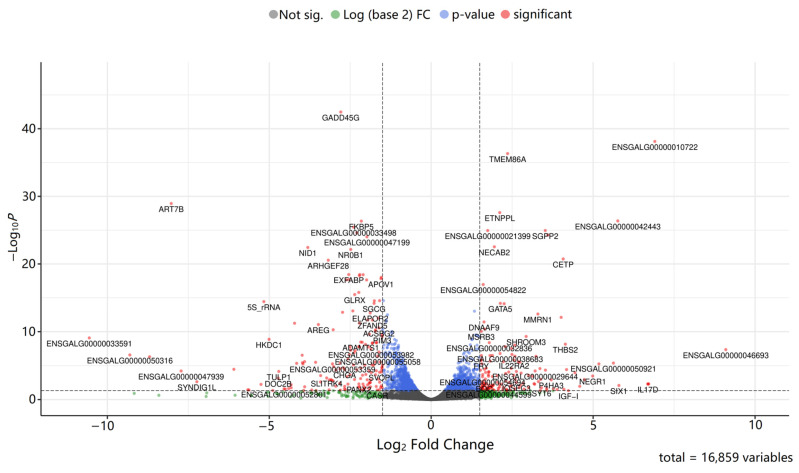
Volcano plot of differentially expressed genes in Group 2 (PGCs) relative to Group 1 (liver cells).

In addition, a volcano plot was constructed to visualize the differential gene expression between groups (Figure 4).

We identified a set of genes whose expression has been previously mentioned in other PGC transcriptomic studies. This includes genes known to be exclusively expressed in PGCs, such as *DMRT1*, *PIWIL1*, *TDRD5*, *MAEL*, *GTSF1*, *SPAG16*, *DNAH1*, *TDRD15*, *MOV10L1*, *FKPB6*, and ENSGALG00000054787 and ENSGALG00000052964 [19]. Our results matched the findings from Jean C. et al. (2015), confirming the expression of *GSTF1*, *DDX4*, *PIWIL1*, *FKBP6*, *DMRT1*, *TDRD5*, *DND1*, and others [27]. Similarities were found with the work of Rengaraj D. et al. (2012) on signaling and metabolic pathways in avian PGCs. We detected the expression of key genes highlighted in the aforementioned study, including *MAD1L1*, *RBL1*, *CCNH*, *WEE1*, *CCNE2*, *DBF4*, *ANAPC10*, *TFDP2*, and *TTK* [28]. Besides the ones mentioned above, our study revealed the expression of genes associated with pluripotency in avian PGCs, namely *SOX10*, *DNMT3B*, *KLF5*, and *VRTN* genes, which are the main pluripotency factors in geese [29]. Finally, we detected the expression of *PRDM14*, a gene with a crucial role in PGC development and specification [30,31].

Of the 16,859 genes analyzed, only 1909 were considered statistically significant after applying the Benjamini–Hochberg correction (padj < 0.05; Table S2). A selection of these genes overlaps with previous PGC studies (Table 2).

**Figure 5:**
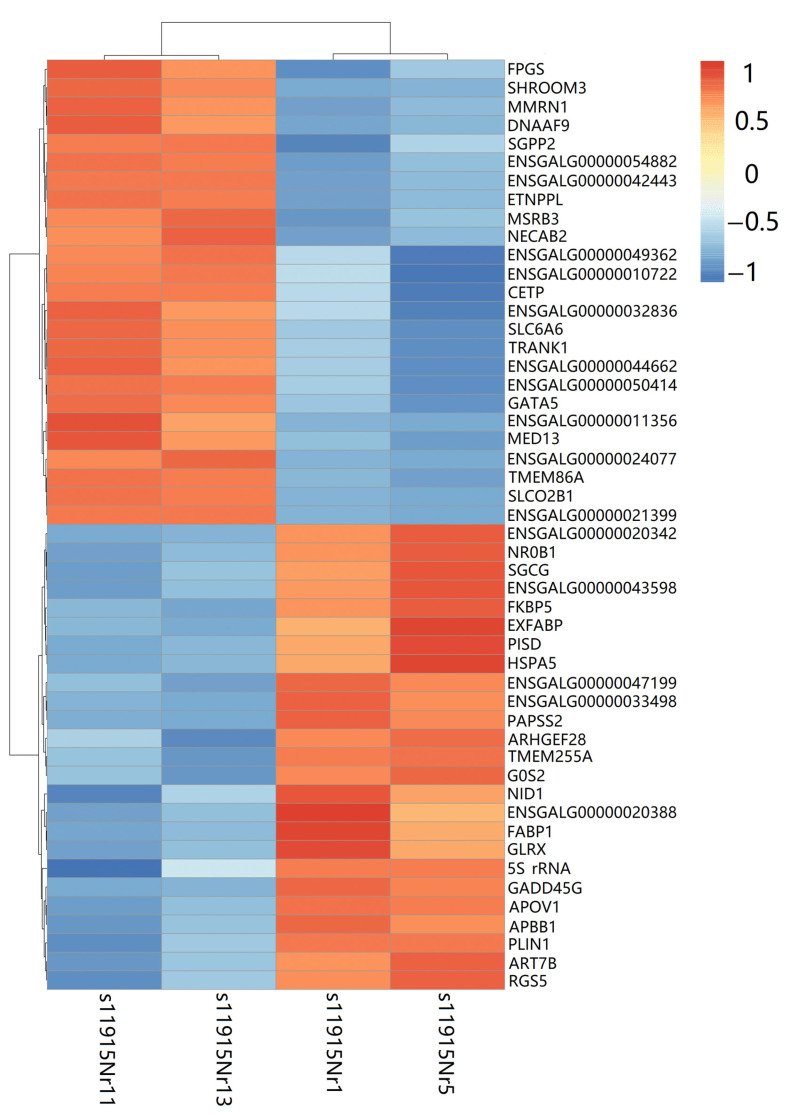
Heatmap with top 25 DEGs for all 4 samples.

Additionally, a heatmap was constructed to display the expression patterns of the top 25 upregulated and downregulated genes between the sample groups (Figure 5).

Upregulated genes in PGCs included: *FPGS*, *SHROOM3*, *MMRN1*, *DNAAF9*, *SGPP2*, *ETNPPL*, *MSRB3*, *NECAB2*, *CETP*, *SLC6A6*, *TRANK1*, *GATA5*, *MED13*, *TMEM86A*, *SLCO2B1*, and genes with unknown functions—ENSGALG00000054822, ENSGALG00000042443, ENSGALG00000049362, ENSGALG00000010722, ENSGALG00000032836, ENSGALG00000044662, ENSGALG00000050414, ENSGALG00000011356, ENSGALG00000024077, and ENSGALG00000021399 (Figure 5). Downregulated in PGCs were the following genes: *NR0B1*, *SGCG*, *FKBP5*, *EXFABP*, *PISD*, *HSPA5*, *PAPSS2*, *ARHGEF28*, *TMEM255A*, *G0S2*, *NID1*, *FABP1*, *GLRX*, 5S_rRNA, *GADD45G*, *APOV1*, *APBB1*, *PLIN1*, *ART7B*, *RGS5*, and other genes with unknown functions—ENSGALG00000020342, ENSGALG00000043598, ENSGALG00000047199, ENSGALG00000033498, and ENSGALG00000020388 (Figure 5).

To elucidate the biological relevance of the 1909 DEGs, we performed the GO enrichment analysis, which covered three blocks: molecular function (MF), biological process (BP), and cellular component (CC). To simplify the result interpretation, data redundancy was minimized, i.e., similar GO terms were reduced, taking into account their hierarchical position and parent nodes (Table 3). The full list of significant GO terms with identifiers and the list of corresponding DEGs are provided in the Supplementary Materials (Table S3).

GO enrichment analysis revealed that DEGs were predominantly associated with protein processing, folding, and trafficking within the endoplasmic reticulum (ER), and translation, ribosome assembly, and mitochondrial respiratory chain function. Key cellular components included the ER, ribosomes, coated vesicles, and respiratory complexes. At the molecular level, terms related to ribosomal structure, ATP-dependent chaperone activity, and isomerase function were most significant. The complete lists of GO terms are provided in Supplementary Materials (Table S3).

## 4. Discussion

A key limitation of our research is the small sample size (*n* = 2 per group), which reduces the statistical power of the differential expression analysis by increasing the risk of false positive discoveries (FDR) and false negatives, where actual DEGs with modest fold changes may remain undetected. We consciously accepted this limitation due to the pilot-scale nature of our work, which aimed to identify the most prominent transcriptomic differences between chicken PGCs and adult liver cells. To mitigate these issues, we used conservative significance thresholds (log_2_ FC > |1.5|and padj < 0.05) as a compensatory measure and focused on functional coherence of the DEG subset with the cell type. Furthermore, the overlap of several DEGs with previously reported PGC markers in other studies provides indirect validation. Therefore, the results presented here should be interpreted as a reliable core signature that highlights the biggest transcriptional differences, rather than as a comprehensive catalog of all DEGs. With this framework in mind, we proceed to discuss our results.

As noted in Table 2, several genes from our DEG set have been previously mentioned in other PGC studies. *NEGR1*, encoding a neuron growth factor, has been proposed as a PGC surface marker [32]. *TDRD15*, *GTSF1*, and novel transcripts ENSGALG00000052964 and ENSGALG00000054787 were also identified as germ-cell-exclusive genes in the study by Doddamani et al. (2023) [19]. The same study reported sex-biased expression for a gene subset. *GOLPH3*, *LIFR*, *DHFR*, *ARHGEF28*, *SIGMAR1*, *GFRA2*, *VCP*, and ENSGALG00000017558 showed increased expression in male PGCs, while ENSGALG00000031327, ENSGALG00000046955, and ENSGALG00000002350 were upregulated in female PGCs. The ENSGALG00000052964 and ENSGALG00000054787 transcripts show homology to *TOPAZ1*, a vertebrate germ cell-specific gene [33,34]. ENSGALG00000031327 encodes a sequence similar to *CHD1W*, a W-chromosome gene that can, therefore, be used for sex identification [52].

Functional analysis of DEGs revealed several biological processes by which they can be grouped.

1. Nervous system development. A notable set of PGC DEGs is associated with nervous system development and function. This may be explained by the fact that both PGCs and neurons are characterized by long-range migration and complex cell–cell communication. Besides *NEGR1*, this set includes *GFRA2*, *NRXN1*, *NCAM1*, *GFRA1*, and *FAM126A*. *GFRA1* and *GFRA2* encode neurotrophin receptors crucial for neuron survival and differentiation [37,53]. *NRXN1* is essential for synapse formation [54], *NCAM1* for synaptic plasticity and neurogenesis [55], and *FAM126A* for the myelination of nerve fibers [45].

2. Lipid metabolism. Other DEG set, including *GOLPH3*, *CYP1A1*, *ALDH1A1*, *CYP2C18*, and *BRCA1*, points to an active role in lipid metabolism. This finding aligns with established evidence that lipid metabolism is tightly coupled with differentiation and energy homeostasis in male germ cells [40].

3. Genome stability. Another set of DEGs, including *VCP*, *PSMC5*, *ATM*, and *BRCA1*, is central to DNA damage response and genome integrity. All of them are necessary for DNA repair: *ATM* acts as a DNA damage sensor [48]; *VCP* facilitates the DNA repair process [56]; *PSMC5* represents a proteasomal component involved in DNA damage response [57]; and *BRCA1* acts as a central coordinator of repair pathways, ubiquitination, and transcription regulation to maintain genomic stability [49,51,58].

A closer investigation of the top 25 upregulated DEGs in PGCs revealed both known and novel genes. Among the annotated genes are *FPGS*, *SHROOM3*, *MMRN1*, *DNAAF9*, *SGPP2*, *ETNPPL*, *MSRB3*, *NECAB2*, *CETP*, *SLC6A6*, *TRANK1*, *GATA5*, *MED13*, *TMEM86A*, and *SLCO2B1*. Novel transcripts with unknown functions include ENSGALG00000054822, ENSGALG00000042443, ENSGALG00000049362, ENSGALG00000010722, ENSGALG00000032836, ENSGALG00000044662, ENSGALG00000050414, ENSGALG00000011356, ENSGALG00000024077, and ENSGALG00000021399, highlighting promising targets for future functional characterization (Figure 5).

These genes can be categorized into several groups.

1. Folate metabolism and nucleotide biosynthesis. Expression of the enzymes *FPGS* and *DHFR*, both essential for folate metabolism, highlights the increased demand for nucleotide precursors in rapidly proliferating PGCs [36,59]. This upregulation is a distinctive feature of cells with high proliferative potential, consistent with the role of *FPGS* in the survival of proliferating cells [59].

2. Lipid metabolism. A cluster of DEGs, including *SGPP2*, *ETNPPL*, *CETP*, and *TMEM86A*, is involved in lipid metabolism. Enzymes coded by *SGPP2*, *ETNPPL*, and *TMEM86A* are essential for processing sphingosine, phosphoethanolamine, and lysoplasmalogens, respectively [60,61,62]. Their substrates and products play crucial roles in myelination, synaptic plasticity, and cell signaling within the nervous system. Specifically, *SGPP2* controls the level of sphingosine-1-phosphate (S1P), a key signaling lipid that regulates neuron migration and survival. The involvement of S1P in critical signaling pathways pinpoints the role of *SGPP2* in ER stress regulation and cell proliferation [60]. *ETNPPL* metabolizes phosphoethanolamine, a precursor of ethanolamine plasmalogens vital for antioxidant protection of the brain and neuronal membranes. This function explains both the significant *ETNPPL* upregulation during embryogenesis [61] and its identification as a primate-specific marker of neural stem cells (NSCs) [63]. Lysoplasmalogens, whose metabolism involves *TMEM86A*, act as membrane stabilizers due to their antioxidant properties and antagonism to lysolecithin [62]. Finally, *CETP* participates in lipid transport, which is consistent with its associations with cholesterol levels [64,65]. By transferring cholesterol esters, *CETP* directly influences the cholesterol availability for membrane construction and repair. In summary, this gene cluster highlights that beyond lipid metabolism, these genes are vital regulators of structural and signaling lipids essential for cellular membranes and neuronal function. The broader list of DEGs further reinforces this, including other lipid-related genes like *GOLPH3*, *CYP1A1*, *ALDH1A1*, and *CYP2C18* [38], highlighting the close relationship between lipid metabolism and cell differentiation and proliferation [40]. Moreover, lipid metabolism is closely related to fertility across humans, animals, and plants [66,67], which explains the significant differential expression of these genes in PGCs—the gamete precursors.

3. Protection and cellular homeostasis. The obtained data suggest that PGC development requires robust mechanisms for cellular stress management. *MSRB3* acts as a key antioxidant enzyme that reduces oxidized proteins and, hence, protects against oxidative stress. Furthermore, it regulates cell proliferation and apoptosis via p53-p21 and p27 pathways [68,69]. Loss of *MSRB3* function causes ER stress and subsequent apoptosis [70]. *NECAB2* maintains both calcium and mitochondrial homeostasis, which is consistent with the mitochondrial dysfunction observed upon its knockout [71]. *SLC6A6* encodes the taurine transporter, which regulates many processes, including cell proliferation, differentiation, calcification, and apoptosis [72]. *SLC6A6* presumably contributes to intracellular signaling via taurine transport [72].

4. Morphogenesis and cell adhesion. Upregulation of the developmental protein, *SHROOM3*, a key regulator of cell shape during morphogenesis [73,74], suggests its potential role in cytoskeletal rearrangement required for PGC migration and tissue integration. *MMRN1*, while known for its role in hemostasis, also participates in cell adhesion [75], potentially facilitating the critical adhesive and signaling interactions between PGCs and their surrounding somatic niche, which are vital for guided migration, survival, and eventual colonization of the gonads. However, we acknowledge that its expression could alternatively reflect a low-level persistence of hematopoietic or endothelial cells from the initial blood isolate. This dual possibility highlights the need for future single-cell resolution studies to definitively assign its expression to PGCs.

5. Transcriptional regulation and development. A subset of genes shows its influence on developmental programs in PGC biology. *GATA5* encodes a transcription factor essential for cell differentiation, particularly during the development of the vertebrate cardiovascular system [76,77]. *MED13*, a component of the transcription coactivator complex, is important for vertebrate embryonic development. Its specific role in zygotic genome activation and successful preimplantation development [78] suggests a fundamental function in establishing totipotency, a key feature of early germ cells. Finally, *SLCO2B1* mediates the cellular uptake of signaling molecules (prostaglandins and steroid conjugates) [79], thus controlling the availability of ligands for nuclear receptors. The latter suggests its indirect effect on transcriptional programs important for cell differentiation and tissue development, making it a potential regulator of PGC maturation or niche communication.

6. Unexplored functions. The last gene subset includes genes with unclear germ cell functions. It includes *DNAAF9*, which encodes an uncharacterized protein with a C-terminal helical region, and the brain-expressed *TRANK1*, the latter being a GWAS candidate for neuropsychiatric disorders [80,81,82].

In summary, the transcriptional signature of the known top 25 DEGs represents PGCs’ highly proliferative cells with adapted metabolic networks, complex stress-response systems, and under precise developmental control, which is consistent with their role as progenitors of the germline.

Novel transcripts were also analyzed, revealing conserved themes in PGC development. Homology searches for the identified transcripts revealed functionally relevant data.

1. Early embryogenesis signaling and morphogenesis. The ENSGALG00000042443 transcript is homologous to *VWDE*, which is implicated in cell proliferation and differentiation [83]. To date, ENSGALG00000010722 has been linked to *SERAF*, a gene highly homologous to *VWDE* [84,85]. Upregulation of *SERAF* is observed in migrating neural crest cells and in Schwann cell precursors during avian embryogenesis [86]. Notably, *SERAF* expression is regulated by *SOX10*, whose expression was detected in our dataset (below the significance threshold). Furthermore, the ENSGALG00000011356 transcript is linked to *ERICH3*, a protein highly expressed in the central nervous system [87], and a component of primary cilia [88]. Cilia coordinate a number of vital signaling pathways (Wnt, TGF-beta, and Shh), which regulate cell fate, differentiation, and migration [89]. The co-expression of *VWDE*/*SERAF* homologs, their regulator *SOX10* [85], and a ciliary component (*ERICH3*) suggests the utilization of evolutionarily conserved molecular programs, namely long-range migration and niche interaction, which are crucial for both neural crest and PGCs.

2. Specialized metabolism and homeostasis. The ENSGALG00000032836 transcript corresponds to *MAOB*, a key enzyme in amine metabolism. *MAOB* regulates neurotransmitter and hormone levels in the central nervous system and peripheral tissues [90,91]. Additionally, ENSGALG00000021399 is linked to *ABCA8*, an ATP-binding cassette transporter that facilitates molecule transport across membranes and contributes to sphingomyelin production in oligodendrocytes, playing a role in myelin formation and maintenance [92]. The upregulation of *MAOB* and *ABCA8* in PGCs pinpoints the necessity for precise control over amine and lipid balance and maintenance of specialized membrane composition during PGC migration and maturation.

3. Immune and hematopoietic pathways. The remaining transcripts are unexpectedly associated with immunity/hematopoiesis. The ENSGALG00000044662 exhibits homology to the *LOC107049909* locus, a member of the leukocyte immunoglobulin-like receptor family, indicating its role in the immune system. The ENSGALG00000024077 transcript is currently linked to *CRYBG3*, a gene implicated in platelet morphology and function [93]. The co-expression of *LOC107049909*/*CRYBG3* may reflect either evolutionarily conserved mechanisms for adhesion, extracellular interaction used by PGC for embryo navigation, or, potentially, a novel immuno-like surveillance or interaction mechanism intrinsic to germline development. The involvement of these genes in PGC development and function requires further research.

## 5. Conclusions

Our comparative transcriptomic analysis of PGCs and adult liver cells, despite the limited sample size (*n* = 2 per group), revealed a core PGC-specific transcriptional signature. It is crucial to acknowledge that liver control represents a heterogeneous cellular mixture, including hepatocytes, epithelial, endothelial and immune cells. This means that the identified DEG set reflects differences between a pure PGC population and an average transcriptional profile of a complex somatic organ. Indeed, this comparison may mask signals specific to individual liver cell subtypes, but it also effectively highlights the global features that fundamentally distinguish PGCs from differentiated somatic tissues. The functional coherence of the identified DEG set with known PGC biology confirms the robustness of this core signature.

The analysis delineated two principal axes of the PGC transcriptome. First, it comprises genes providing the fundamental properties of embryonic progenitors: hyperproliferation (folate metabolism, *FPGS* and *DHFR*), specialized metabolic and membrane homeostasis (lipid transport, *CETP* and *TMEM86A*; detoxification, *MAOB*; and antioxidant defense, *MSRB3*), and genome stability maintenance (*BRCA1* and *ATM*). This profile stands in sharp contrast to the metabolic function dominant in differentiated liver cells. Second, unexpectedly, a profound molecular convergence between PGCs and neural crest cells was discovered. This is evidenced by the active expression of a suite of genes governing long-range migration (*SHROOM3* and *MMRN1*), neuronal signaling and adhesion (*NEGR1*, *GFRA1/2*, and *NCAM1*), and lipid metabolism critical for myelination and synaptic plasticity (*SGPP2* and *ETNPPL*). This finding suggests that PGCs, like neural crest cells, recruit evolutionarily conserved modules to accomplish similar morphogenetic tasks. The detection of this shared program against a heterogeneous control background underscores its specificity to these migratory cell types. Thus, despite the methodological constraints, our study demonstrates that PGCs are not merely proliferating cells but a highly specialized population with a unique “neuro-lipid” metabolic profile and complex developmental systems ensuring their migration and fate determination. The unannotated transcripts related to immune pathways and primary cilia signaling (*ERICH3*), identified here, point to promising new avenues for the functional characterization of avian PGC biology. While our comparative approach provides a robust core signature, future studies using single-cell RNA-seq or fluorescence-activated cell sorting (FACS) with specific surface markers will be essential to definitively assign all of the identified genes to PGCs.

## Figures and Tables

**Table 1:** Number of sequenced fragments and bases (RNA).

Group	Cell Type	ID	Number of Fragments (In Million)	Number of Bases (In Gb)
1	Liver cells	S11915Nr1	53.066	10.702
		S11915Nr5	67.045	13.504
2	PGCs	S11915Nr11	58.034	11.698
		S11915Nr13	58.202	11.740

**Table 2:** List of significant differentially expressed genes with known association to PGCs.

Gene ID	Gene	GGA	padj *	Source	Description
ENSGALG00000002350	ENSGALG00000002350	14	0.0029	Doddamani D. et al., 2023 [19]	*DNAH3* gene ortholog. *DNAH3* encodes a motor protein involved in sperm motility; associated with male infertility.
ENSGALG00000046955	ENSGALG00000046955	-	0.0175	Doddamani D. et al., 2023 [19]	*DNAH3* gene ortholog.
ENSGALG00000031327	ENSGALG00000031327	W	8.8415 × 10^−5^	Doddamani D. et al., 2023 [19]	Encodes a sequence similar to *LOC374195* and *CHD1W*. *CHD1W* is located on the W chromosome in female birds and can be used to determine sex (homolog on Z—*CHD1Z*).
ENSGALG00000011350	*NEGR1*	8	0.0003	Kim J.L. et al., 2024 [32]; Jean C. et al., 2015 [27]	Encodes a neuron growth factor involved in neuron growth and cell adhesion. Proposed as a surface marker for PGCs in chickens and zebrafish [32].
ENSGALG00000052964	ENSGALG00000052964	2	0.0003	Doddamani D. et al., 2023 [19]	Encodes a sequence close to the *TOPAZ1* gene, which is necessary for normal spermatogenesis and male fertility. Specific to vertebrate germ cells [33,34].
ENSGALG00000000349	*PSMC5*	27	0.0019	Doddamani D. et al., 2023 [19]	Component of the 26S proteasome. Involved in cell cycle progression, apoptosis, and DNA repair.
ENSGALG00000054787	ENSGALG00000054787	2	0.0067	Doddamani D. et al., 2023 [19]	*TOPAZ1* transcript variant, mRNA
ENSGALG00000016492	*TDRD15*	3	0.0093	Doddamani D. et al., 2023 [19]	Associated with spermatogenesis [35].
ENSGALG00000001109	*GTSF1*	27	0.0124	Doddamani D. et al., 2023; [19] Jean C. et al., 2015 [27]	Participates in cell differentiation and spermatogenesis.
ENSGALG00000026757	*DHFR*	Z	0.0027	Doddamani D. et al., 2023 [19]	A key enzyme in folate metabolism [36].
ENSGALG00000003733	*LIFR*	Z	0.0040	Doddamani D. et al., 2023 [19]	A member of the cytokine receptor I family, involved in cell differentiation, proliferation, and survival in adult organisms and embryos.
ENSGALG00000032856	*GFRA2*	22	0.0089	Doddamani D. et al., 2023 [19]	Receptor of neurotrophin, a growth factor that plays an important role in the development and survival of sympathetic neurons [37].
ENSGALG00000028996	*SIGMAR1*	Z	0.0242	Doddamani D. et al., 2023 [19]	Encodes a receptor that plays an important role in cellular functions of various tissues associated with the endocrine, immune, and nervous systems.
ENSGALG00000001986	*VCP*	Z	0.0286	Doddamani D. et al., 2023 [19]	Plays an important role in protein degradation, intracellular membrane fusion, DNA repair and replication, cell cycle regulation, and NF-kB pathway activation.
ENSGALG00000028267	*GOLPH3*	Z	0.0347	Doddamani D. et al., 2023 [19]	Plays a regulatory role in Golgi transport. Regulates cellular sphingolipids, thereby promoting the transmission of growth factor signals and cell proliferation [38].
ENSGALG00000014923	*ARHGEF28*	Z	2.7824 × 10^−21^	Doddamani D. et al., 2023 [19]	Functions as a RhoA-specific guanine nucleotide exchange factor that regulates signaling pathways downstream of integrins and growth factor receptors.
ENSGALG00000017558	ENSGALG00000017558	Z	3.1608 × 10^−6^	Doddamani D. et al., 2023 [19]	Encodes a sequence similar to the uncharacterized locus *LOC768709*.
ENSGALG00000001325	*CYP1A1*	10	0.0075	Li D. et al., 2017 [39]; Zuo Q. et al., 2015 [40]	Encodes a cytochrome P450 monooxygenase involved in the metabolism of fatty acids, steroid hormones, and vitamins [41].
ENSGALG00000015147	*ALDH1A1*	Z	0.0009	Zuo Q. et al., 2015 [40]	Encodes a cytosolic dehydrogenase involved in lipid metabolism and retinol metabolism.
ENSGALG00000052243	*CYP2C18*	6	0.0012	Zuo Q. et al., 2015 [40]	Encodes a cytochrome P450 monooxygenase involved in retinoid metabolism.
ENSGALG00000009107	*NRXN1*	3	0.0243	Jean C. et al., 2015 [27]	Neurexin is a membrane protein that plays an important role in the formation and functioning of synapses in the nervous system.
ENSGALG00000009495	*FGFR2*	6	0.0009	Jean C. et al., 2015 [27]	Tyrosine kinase, which acts as a receptor for fibroblast growth factors, plays an important role in regulating cell proliferation, differentiation, migration, and apoptosis, and in regulating embryonic development.
ENSGALG00000027240	*TSPAN13*	2	0.0376	Jean C. et al., 2015 [27]	Member of tetraspanin family, which mediates signal transduction events important for the regulation of cell development, activation, growth, and motility.
ENSGALG00000038740	*AMY2A*	8	0.0332	Jean C. et al., 2015 [27]	An amylase isoenzyme is involved in carbohydrate metabolism.
ENSGALG00000007839	*NCAM1*	24	0.0356	Jean C. et al., 2015 [27]	Cell adhesion protein of the immunoglobulin superfamily. Participates in cell–cell and cell–extracellular matrix interactions during development and differentiation. Plays a role in the development of the nervous system by regulating neurogenesis, neurite growth, and cell migration.
ENSGALG00000005451	*HELLS*	6	0.0235	Jean C. et al., 2015 [27]	Lymphoid helicase. Developmental protein involved in cell proliferation.
ENSGALG00000011190	*PLAC8*	4	0.0013	Jean C. et al., 2015 [27]	Encodes a protein involved in embryonic development [42]. Involved in the development of internal organs [43].
ENSGALG00000009173	*GFRA1*	6	0.0071	Jean C. et al., 2015 [27]	Receptor for alpha 1 factor associated with glial cell-derived neurotrophic factor (GDNF). Plays a key role in controlling neuron survival and differentiation.
ENSGALG00000016362	*SH3YL1*	3	0.0462	Jean C. et al., 2015 [27]	Encodes an androgen receptor involved in meiosis, cell migration, and hair follicle development [44].
ENSGALG00000032386	*DCLK2*	4	3.0129 × 10^−8^	Jean C. et al., 2015 [27]	Encodes a member of the protein kinase superfamily and the doublecortin family.
ENSGALG00000010925	*FAM126A*	2	0.0399	Jean C. et al., 2015 [27]	Component of the complex that acts as a regulator of phosphatidylinositol 4-phosphate synthesis. Participates in the myelination of the central and peripheral nervous systems [45].
ENSGALG00000015136	*ILDR1*	1	0.0311	Jean C. et al., 2015 [27]	Multidimensional receptor on the cell surface. Supports epithelial barrier function [46].
ENSGALG00000017159	*ATM*	1	0.0034	Rengaraj D. et al., 2022 [47]	Serine/threonine protein kinase, necessary for cellular response to DNA damage and genome stability [48].
ENSGALG00000002781	*BRCA1*	27	0.0002	Rengaraj D. et al., 2022 [47]	Encodes a E3 ubiquitin–protein ligase that plays an important role in DNA repair and genome stability [49]. Acts as a transcription activator [50]. Involved in lipid metabolism [51].

* padj—adjusted *p*-value calculated with Benjamini–Hochberg correction.

**Table 3:** Statistics of GO terms identified for significant DEGs.

Significant GO Terms					
Before reduction			After reduction		
BP	MF	CC	BP	MF	CC
35	7	42	14	7	15

Note: BP—biological process; MF—molecular function; CC—cellular component.

## Data Availability

The data presented in this study are included in the article and the Supplementary Materials. Further inquiries can be directed to the corresponding author(s).

## Supplementary Materials

The following supporting information can be downloaded at https://www.mdpi.com/article/10.3390/ani16030522/s1. Table S1: List of all 16,859 analyzed differentially expressed genes.; Table S2: List of statistically significant (1909) genes after applying the Benjamini–Hochberg correction; Table S3: List of reduced GO-terms with identifiers and identified DEGs associated with them.

## Abbreviations

The following abbreviations are used in this manuscript:

PGC	Primordial germ cell
DEG	Differentially expressed gene
GE	Genome editing
CCU	Center of collective use
GO	Gene ontology
MF	Molecular function
BP	Biological process
CC	Cellular component
ER	Endoplasmic reticulum
NSC	Neural stem cell
IEE	Inner enamel epithelium
GWAS	Genome-wide association study
FACS	fluorescence-activated cell sorting
FDR	False discovery rate
